# Patterns of Technology Use in Patients Attending a Cardiopulmonary Outpatient Clinic: A Self-Report Survey

**DOI:** 10.2196/ijmr.3955

**Published:** 2015-03-06

**Authors:** Rebecca T Disler, Sally C Inglis, Phillip J Newton, David C Currow, Peter S Macdonald, Allan R Glanville, DorAnne Donesky, Virginia Carrieri-Kohlman, Patricia M Davidson

**Affiliations:** ^1^Centre for Cardiovascular and Chronic CareFaculty of HealthUniversity of Technology, SydneyBroadwayAustralia; ^2^Discipline Palliative and Supportive ServicesFlinders UniversityAdelaideAustralia; ^3^St Vincent's Hospital and Victor Chang Cardiac Research InstituteSydneyAustralia; ^4^St Vincent's HospitalSydneyAustralia; ^5^Physiological NursingUniversity of CaliforniaSan Francisco, CAUnited States; ^6^Department of Acute and Chronic CareJohns Hopkins UniversityBaltimore, MDUnited States

**Keywords:** chronic obstructive pulmonary disease, chronic disease, self-management, self-care, telemedicine, eHealth, mHealth

## Abstract

**Background:**

Self-management education for cardiopulmonary diseases is primarily provided through time-limited, face-to-face programs, with access limited to a small percentage of patients. Telecommunication tools will increasingly be an important component of future health care delivery.

**Objective:**

The purpose of this study was to describe the patterns of technology use in patients attending a cardiopulmonary clinic in an academic medical center.

**Methods:**

A prevalence survey was developed to collect data on participant demographics (age in years, sex, and socioeconomic status); access to computers, Internet, and mobile phones; and use of current online health support sites or programs. Surveys were offered by reception staff to all patients attending the outpatient clinic.

**Results:**

A total of 123 surveys were collected between March and April 2014. Technological devices were a pervasive part of everyday life with respondents engaged in regular computer (102/123, 82.9%), mobile telephone (115/117, 98.3%), and Internet (104/121, 86.0%) use. Emailing (101/121, 83.4%), researching and reading news articles (93/121, 76.9%), social media (71/121, 58.7%), and day-to-day activities (65/121, 53.7%) were the most common telecommunication activities. The majority of respondents reported that access to health support programs and assistance through the Internet (82/111, 73.9%) would be of use, with benefits reported as better understanding of health information (16/111, 22.5%), avoidance of difficult travel requirements and time-consuming face-to-face appointments (13/111, 18.3%), convenient and easily accessible help and information (12/111, 16.9%), and access to peer support and sharing (9/111, 12.7%). The majority of patients did not have concerns over participating in the online environment (87/111, 78.4%); the few concerns noted related to privacy and security (10/15), information accuracy (2/15), and computer literacy and access (2/15).

**Conclusions:**

Chronic disease burden and long-term self-management tasks provide a compelling argument for accessible and convenient avenues to obtaining ongoing treatment and peer support. Online access to health support programs and assistance was reported as useful and perceived as providing convenient, timely, and easily accessible health support and information. Distance from the health care facility and a lack of information provision through traditional health sources were both barriers and enablers to telehealth. This is particularly important in the context of a cardiopulmonary clinic that attracts patients from a large geographical area, and in patients who are most likely to have high health care utilization needs in the future. Telecommunication interfaces will be an increasingly important adjunct to traditional forms of health care delivery.

## Introduction

The increasing burden of non-communicable diseases, such as heart and respiratory disease, is placing increasing pressure on global health systems [1,2]. The incidence and cumulative burden of these chronic progressive disorders is accentuated through population aging [1]. The prevalence of chronic heart failure (CHF) is 23 million worldwide with an overall prevalence of 2-3% of the population in the United States and Europe [2-5]. The global prevalence of chronic obstructive pulmonary disease (COPD) is estimated at 65 million and COPD is now responsible for 5% of all deaths globally [6,7]. Despite optimal pharmacological and medical treatments, individuals with COPD and CHF continue to experience high symptom burden, most commonly dyspnea and fatigue [8-12]. Both COPD and CHF are frequent causes of hospitalization and require self-management strategies [8-12]. The economic costs of COPD are approximately US$ 40 billion annually and this financial burden will only increase [8-12]. Daily symptoms, poor physical functioning, progressive social isolation, and caregiver burden contribute to this disease burden [6,7,13].

The burden of non-communicable diseases extends over time and the life course [14,15]. Self-management education for people with COPD and CHF, including symptom management strategies, exercise, and reinforcement of activity and medication adherence, are primarily provided through pulmonary and cardiac rehabilitation and heart failure specific disease management programs [16-20]. These interventions are commonly episodic, of short duration, and available only to a small percentage of individuals [16,17], with access limited by functional debilitation associated with chronic illness [21,22]. Although discrete disease management strategies are an integral element of evidence-based care, it is increasingly apparent that there are some symptom management issues that are germane across chronic conditions [23]. Self-management support should be targeted through multiple modes of delivery with a broad-based symptom focus [24]. Although chronic conditions such as COPD and CHF have received greater attention from the medical community over the past decades, the burden of disease at an individual level is less well recognized [25].

The most effective and economically sustainable approaches to support patients with chronic illnesses such as COPD and CHF, beyond acute exacerbations, require future investigation [13,18,24,26-28]. The disease burden and long-term tasks of self-management that confront patients are a compelling argument for accessible and convenient avenues to obtaining ongoing treatment and peer support [29-31]. Access to Web-based health information and support is well established in the United States with a recent report noting that over 50% of adults aged over 65 years use the Internet [28,32-35]; however, internationally, use is not so widespread [28,30-36]. In the Australian context, studies have explored Web-based health interventions, but there is limited information as to the patterns of technology use in this particular patient group [37].

Although technology access is challenging for some older adults who are the most burdened with chronic conditions, communication tools have become a critical component of health care delivery [29-31]. Rapid advances in tools that provide instant access to health information and rich resources for self-care have already created paradigm shifts in health consumer attitudes about their health and health care [28-31]. The evolution of eHealth (health care delivery through Internet and telehealth communications for surveillance, health promotion, and symptom or disease management) and the introduction of mHealth (monitoring, personal digital assistants, and other wireless devices) are markedly altering the collaboration and interaction between consumers, health providers, and institutions [38-40]. Asynchronous forms of health interaction, such as through email or discussion boards, allow individuals to receive self-management and condition support by posing questions to their provider without having to establish a formal face-to-face consultation (synchronous interaction) [36,41]. These converging factors will shape the development and testing of future interventions aimed at improving health outcomes and reducing costs across chronic illnesses. The new generation of empowered health consumers will expect that health care systems accommodate their changing needs and preferences for how they receive care, including access to evidence-based therapies [28-31].

In order to determine the future feasibility of Internet-based health care delivery, the reported prevalence study was undertaken to describe the patterns of technology use in patients attending a cardiopulmonary clinic in an academic medical center. The cardiopulmonary patients responding to this survey have provided a sample of those individuals most likely to have high health care utilization needs in the future; it is important to take this initial step in understanding whether these consumers are technology ready [42-44].

## Methods

### Objective

The objective of the study was to describe the prevalence and patterns of technology use in patients attending a cardiopulmonary outpatient clinic through a self-report survey.

### Recruitment

Patients attending a cardiopulmonary outpatient clinic at an academic medical center were invited to participate in this anonymous survey. All patients attending the cardiopulmonary outpatient clinic were eligible to participate.

The cardiopulmonary clinic is located within an academic medical center and provides services for patients with a variety of conditions including COPD, CHF, advanced lung disease, heart transplantation, and pulmonary hypertension. This clinic is a central referral setting for surrounding regional areas; consequently, individuals travel from all areas within the state to access specialist treatment.

### Instrument

A prevalence survey was developed in consultation with experts in the field of chronic illness and Internet-based health care delivery. The survey was presented in four sections with 11 questions used to capture information on participant demographics (age in years, sex, socioeconomic status); access and use of computers, Internet, and mobile telephones; and currently accessed health support sites. Socioeconomic status was described using the Australian socioeconomic indexes for areas (SEIFA) [45]. These indices summarize “the relative socioeconomic advantage and disadvantage of areas using data from the Census of Population and Housing” and are reported through Australian area postcode (area zip code). Indices are based on a number of variables including employment, private and rented occupied housing, family makeup, and highest qualification, to name a few [45]. Nominal tick boxes and free text short answer questions were used to collect responses. Respondents were able to give multiple answers to appropriate nominal and free text questions, noted by “please tick all that apply”.

The survey was piloted for 1 week in the cardiopulmonary clinic and 10 surveys were checked for completion and coherence with the questions asked prior to continuing with data collection. There was limited missing data in this initial phase, however, “Please turn over” was added to the bottom of the page for ease. No other adjustments were required. The final survey contained four sections with 11 questions and took approximately 5-10 minutes to complete (see Multimedia Appendix 1).

### Data Collection

Surveys were offered by reception staff to all patients attending the outpatient clinic at appointment registration. Surveys on clipboards were also placed on tables within the waiting area for patients to complete as they wished. Participation was voluntary with hard copy surveys completed and placed anonymously in a sealed submission box within the waiting area.

### Data Analysis

Descriptive statistics were used to analyze all aspects of the survey data.

### Ethical Issues

Ethical approval was provided by the collaborating academic institution and clinical site; approval numbers LHR/13/SVH/5 and 2012-149A. Participation was voluntary and anonymous.

## Results

### Respondents

A total of 123 surveys were collected between March and April 2014. Approximately 543 patients attended appointments at the cardiopulmonary clinic during the study period, resulting in an overall response rate of 22.7%. The overwhelming majority of respondents completed the survey questions in full. This took into consideration respondents who answered “no” to regular computer or Internet who were precluded from completing particular subsequent questions; all previous responses from these respondents were included in the descriptive statistics. All 123 respondents answered questions in regards to gender, with more females (72/123, 58.5%) noted to have completed the surveys than males (51/123, 41.5%). Age was reported in 118 of 123 (95.9%) surveys with median respondent age of 56 years (range 18-77), and 52.5% (62/118) of respondents aged between 50 and 64 years. All respondents noted their area zip code and from this just under half (55/123, 44.7%) of the respondents were considered to live in middle socioeconomic areas with under one-third coming from low socioeconomic areas (32/123, 26.0%) and under one-third living in high socioeconomic areas (36/123, 29.3%) (Table 1).

**Table 1:** Respondent demographic characteristics.

Descriptive characteristics		n (%)
**Gender (n=123)**		
	Male	51 (41.5)
	Female	72 (58.5)
**Age, years (n=118)**		
	Median (range)	56 (18-77)
	Under 50	42 (35.6)
	50-64	62 (52.5)
	Over 65	14 (11.9)
**Socioeconomic indexes for areas based on postcode (SEIFA), Australia, 2011** ^a^ **(n=123)**		
	Low income (Deciles 1 and 2)	32 (26.0)
	Middle income (Deciles 3 to 8)	55 (44.7)
	High income (Deciles 9 and 10)	36 (29.3)

^a^Australian Bureau of Statistics. Socioeconomic indexes for areas: robustness, diversity within larger areas, and the new geography standard Commonwealth of Australia 2012; ABS Catalogue no. 1351.0.55.038.

### Computer Use

All 123 respondents answered questions related to computer use with the majority of respondents engaged in regular computer use (102/123, 82.9%), defined as more than four times per week. The overwhelming majority had access to a device at home (118/123, 95.9%) mainly in the form of a laptop (91/123, 77.1%); however, over half additionally had access to a desktop (60/123, 50.8%) and a tablet (60/123, 50.8%). Fewer than half the respondents had access to a computer at work for personal use (58/123, 47.2%) and in most cases this access was a desktop computer (44/58, 75.9%). There was no marked difference in computer use across age groups or gender; however, respondents who came from lower socioeconomic areas (32/123, 26.0%) noted less regular computer use (24/32, 75%) compared with other groups (47/55, 85% in middle and 31/36, 86% in high socioeconomic groups) (Table 2).

**Table 2:** Questions relating to access and use of technology.

Access to technology		n (%)^a^
**Regular computer use (n=123)**		
	Yes	102 (82.9)
	No	21 (17.1)
**Regular computer use, “yes”, by age group, years (n=118)**		
	Under 50 (n=42)	33 (78.6)
	50 – 65 (n=62)	53 (85.5)
	65 and over (n=14)	11 (78.6)
**Regular computer use, “yes”, by socioeconomic area** ^b^ **(n=123)**		
	Low socioeconomic area (n=32)	24 (75.0)
	Middle socioeconomic area (n=55)	47 (85.5)
	High socioeconomic area (n=36)	36 (86.1)
**Access to a computer device at home (n=118)**		
	Desktop	60 (50.8)
	Laptop	91 (77.1)
	Tablet	60 (50.8)
**Access to a computer device through work (n=58)**		
	Desktop	44 (75.9)
	Laptop	31 (53.4)
	Tablet	17 (29.3)
**Regular Internet use (n=121) **		
	Yes	104 (86.0)
	No	17 (14.0)
**Regular Internet use, “yes”, by age group, years (n=116)**		
	Under 50 (n=41)	37 (90.2)
	50 – 65 (n=62)	52 (83.9)
	65 and over (n=13)	11 (84.6)
**Regular Internet use, “yes”, by socioeconomic area** ^b^ **(n=121)**		
	Low socioeconomic area (n=32)	25 (78.1)
	Middle socioeconomic area (n=54)	48 (88.9)
	High socioeconomic area (n=35)	31 (88.6)
**Mode of Internet access at home (n=120)**		
	Yes	113 (94.2)
	No	7 (5.8)
**If yes to home Internet access, (n=110)**		
	Wireless	61 (55.5)
	Broadband	34 (30.9)
	Cable/DSL/fiber	9 (8.2)
	Dial-up	2 (1.8)
	Unsure	4 (3.6)
**Mode of Internet access outside the home (n=120)**		
	Yes	81 (67.5)
	No	39 (32.5)
**If yes to Internet access outside the home (n=93)**		
	At work	62 (66.7)
	Via public wireless	58 (62.4)
	Via smartphone	31 (33.3)
	Via friend’s place	24 (25.8)
	Via Internet café	10 (10.8)
**Key Internet activities (n=121) **		
	Emailing	101 (83.4)
	Browsing, researching, reading news articles	93 (76.9)
	Social media	71 (58.7)
	Day to day activities (shopping, banking, and browsing)	65 (53.7)
	Browsing for health information	56 (46.3)
	Skype or video calls	36 (29.8)
**Access to a mobile phone (n=117)**		
	Yes	115 (98.3)
	No	2 (1.7)
**Key mobile phone activities (n=115) **		
	Phone calls	111 (96.5)
	Sending texts	100 (86.9)
	Internet browsing	62 (53.9)
	Checking and sending emails	57 (49.5)
	Other (playing games, social media, apps)	6 (5.3)

^a^Multiple responses to questions were accepted in free text questions and respondents were instructed to “tick all that apply” when responding to nominal questions. In this context, the sum of percentages will be more than 100%.

^b^Australian Bureau of Statistics. Socioeconomic indexes for areas: robustness, diversity within larger areas, and the new geography standard Commonwealth of Australia 2012; ABS Catalogue no. 1351.0.55.038

### Mobile Phone Use and Activities

The majority (117/123, 95.1%) of respondents answered questions related to mobile phone use and activities, with all but two respondents reporting that they used a mobile phone (115/117, 98.3%). Phone calls (111/115, 96.5%) and sending texts (100/115, 86.9%) were the two main activities carried out using a mobile phone. Over half of the respondents additionally used their phone for Internet browsing (62/117, 53.0%) and half for checking and sending emails (57/115, 49.5%) (Table 2).

### Internet Use and Activities

The majority (121/123, 98.4%) of respondents answered questions related to Internet use and activities, with the majority reporting regular Internet use (104/121, 86.0%). Internet use did not differ across age or gender; however, similar to computer use, those from lower socioeconomic areas had a reduced regular Internet use (25/32, 78%). Internet in the home setting was accessed by 94.2% (113/120) of respondents and in the main this was through wireless (61/110, 55.5%) or through broadband access (34/110, 30.9%). The majority of respondents also reported access to the Internet outside the home (81/120, 67.5%) and this was accessed either at work in line with computer access above (62/93, 67%) or through public wireless (58/93, 62%). A further third of individuals additionally had access to the Internet through smartphones (31/93, 33%) and others had access through a friend’s home (24/93, 26%) and Internet cafes (10/93, 11%) (Table 2).

The main activities undertaken through an Internet platform were reported in 121 of 123 (98.4%) of respondents with emailing (101/121, 83.4%), browsing, researching, and reading news articles (93/121, 76.9%), accessing social media (71/121, 58.7%), and day-to-day activities including online shopping, banking, and general browsing (65/121, 53.7%), as the most common. Just under half of respondents (56/121, 46.3%) used the Internet to browse health information and under a third (36/121, 29.8%) for Skype and video calling (Table 2). More female respondents noted that they used the Internet for both social media (female 50/69, 72% vs male 21/47, 45%,) and daily activities including online shopping banking and browsing (female 42/69, 61%, vs male 23/47, 49%) compared with their male counterparts. In regard to socioeconomic status, those from higher income areas showed a higher rate of email (32/34, 94% vs 43/51, 84% and 26/31, 84% in middle and lower socioeconomic groups respectively), research and reading the news (30/34, 88% vs 39/51, 76% in middle and 39/51, 77% in lower socioeconomic groups), and accessing health information through the Internet (21/34, 62% vs 23/51, 45% in middle and 12/31, 39% in lower socioeconomic groups). Those respondents from middle socioeconomic areas were more likely to access social media (37/51, 73%) compared with the other groups (17/34, 50% in higher and 17/31, 55% in lower socioeconomic groups). Respondents from lower socioeconomic areas were additionally less likely to Skype (6/31, 19% vs 12/34, 35% in higher and 18/51, 35% in lower socioeconomic groups) or engage in daily online activities, such as shopping, banking, and browsing (11/31, 35% vs 23/34, 86% in higher and 31/51, 61% in lower socioeconomic groups) (Table 2).

### The Potential for Web-Based Support and Information

The majority of respondents (111/123, 90.2%) answered questions in relation to access, concerns, and use of technology-based health websites. The majority answered that they would find it useful to have access to support programs and assistance with health problems through the Internet (82/111, 73.9%). Respondents between the ages of 50 to 65 years had a slightly higher positive response to this (45/54, 83%) compared with those in the under 50 years group (25/40, 63%) and the over 65 years group (8/12, 67%). Interestingly, those from higher socioeconomic areas were less likely to respond positively to finding benefit from online support and information, with only 67% (22/33) responding “yes”, compared with 75% (21/28) in lower socioeconomic areas and 78% (39/50) in middle socioeconomic areas. The majority of respondents gave reasons as to why they would access online support (71/111, 63.9%) with the main reasons being: better able to understand health information and condition management (16/71, 23%), avoid difficult travel requirements and time-consuming face-to-face appointments (13/71, 18%), and have convenient and easily accessible help and information (12/71, 17%). Nine (13%) of 71 reported “the more help the better” or words to that effect, and nine (13%) of 71 noted the benefit of peer support and sharing. It is also important to note that six (8%) of 17 respondents wrote that online information would address the difficulty they experienced in accessing information from their health providers and a further six (8%) of 71 noted that they would be able to get up-to-date advice on management and treatments (Table 3).

**Table 3:** Questions regarding online access, concerns, and currently used sites.

Online access, concerns and currently accessed sites			n (%)^a^
**Would you find it useful to be able to access support programs using the Internet to assist you with your health problems? (n=111)**			
	Yes		82 (73.9)
	No		29 (26.1)
**Would you find access through Internet useful, “yes”, by age group, years (n=106)**			
	Under 50 (n=40)		25 (62.5)
	50 – 65 (n=54)		45 (83.3)
	65 and over (n=12)		8 (66.7)
**Would you find access through Internet useful, “yes”, by socioeconomic area** ^b^ **(n=111)**			
	Low socioeconomic area (n=28)		21 (75.0)
	Middle socioeconomic area (n=50)		39 (78.0)
	High socioeconomic area (n=33)		22 (66.7)
**Reported reasons (n=71)**			
	Better understanding of health information and condition management		16 (22.5)
	Avoid difficult travel requirements and less time consuming		13 (18.3)
	Convenient and accessible help and information		12 (16.9)
	“The more help the better”		9 (12.7)
	Peer support and sharing		9 (12.7)
	Address difficulty in accessing disease information from health providers		6 (8.5)
	Up-to-date advice on management and treatments		6 (8.5)
**Are there health education or social group sites on the Internet that you have found helpful? (n=112)**			
	Yes		60 (53.6)
	No		52 (46.4)
**Health education or social group sites helpful, “yes”, by age group, years (n=107)**			
	Under 50 (n=38)		20 (52.6)
	50 – 65 (n=57)		29 (50.9)
	65 and over (n=12)		7 (58.3)
**Health education or social group sites helpful, “yes”, by socioeconomic area** ^b^ **(n=112)**			
	Low socioeconomic area (n=30)		14 (46.7)
	Middle socioeconomic area (n=48)		27 (56.3)
	High socioeconomic area (n=34)		19 (55.9)
**Reported health education or social group sites (n=52)**			
	Health organization or research sites		16 (31)
		Australian Heart/Lung Transplant Association	7 (43.8)
		Diabetes	3 (18.8)
		Heart Lung Transplant Network	1 (6.3)
		Arthritis Australia	1 (6.3)
		Cystic Fibrosis	1 (6.3)
		Hemochromatosis organization	1 (6.3)
		Heart and lung sites	1 (6.3)
		Heart foundation	1 (6.3)
	General browsing for health information and education		13 (25.0)
	Medication and treatment information and side effects		9 (17.9)
	Facebook for disease-specific support groups		8 (15.4)
	Donate Life		1 (1.9)
	Health rebate and concession information		1 (1.9)
	Online mental health programs (Sadness and Depression program)		1 (1.9)
**Would you have any concerns about participating in support programs via the Internet? (n=111)**			
	Yes		24 (21.6)
	No		87 (78.4)
**Concern about participating, “yes”, by age group, years (n=106)**			
	Under 50 (n=41)		8 (19.5)
	50 – 65 (n=53)		12 (22.6)
	65 and over (n=12)		3 (25.0)
**Would you find access through Internet useful, “yes”, by socioeconomic area** ^b^ **(n=111)**			
	Low socioeconomic area (n=28)		5 (17.9)
	Middle socioeconomic area (n=50)		13 (26.0)
	High socioeconomic area (n=33)		6 (18.2)
**Reported concerns (n=15)**			
	Privacy and security		10 (66.7)
	Accuracy of information		2 (13.3)
	Computer literacy and access		2 (13.3)
	Limited Australian-based sites		1 (6.7)
	Misinterpretation of information		1 (6.7)
	No support group for my condition		1 (6.7)

^a^Multiple responses to questions were accepted in free text questions and respondents were instructed to “tick all that apply” when responding to nominal questions. In this context, the sum of percentages will be more than 100%.

^b^Australian Bureau of Statistics. Socioeconomic indexes for areas: robustness, diversity within larger areas, and the new geography standard Commonwealth of Australia 2012; ABS Catalogue no. 1351.0.55.038.

### Health Information and Education Websites Currently Accessed

The majority of respondents answered questions relating to health information and education sites currently accessed through the Internet (112/123, 91.1%). Over half of the respondents were already accessing websites that they felt were useful (60/112, 53.6%) and this was marginally higher in those aged 65 years and above (7/12, 58%) than those from middle (29/57, 51%) and low socioeconomic areas (20/38, 53%). A total of 52 (46.4%) of the 112 respondents reported commonly accessed sites, with health organizations and research sites (16/52, 31%), including Australian Heart/Lung Transplant Association, most common. One-third (16/52, 31%) of respondents stated that they did not access a particular website, but that they generally browsed the Internet for health information and education, with a further nine (17%) of 52 respondents accessing sites for medication and treatment information specifically. Eight (15%) of 52 responded that they accessed disease-specific Facebook support groups, and single individuals noted they accessed Donate Life, health rebate and concession sites, and an online mental health support program run by the academic medical center itself (1/52, 2%, respectively) (Table 3).

### Concerns Over Accessing Information and Support Online

When asked if respondents had concerns over accessing and participating in online support programs, the overwhelming majority of respondents answered the question (111/123, 90.2%) and did not have concerns (87/111, 78.4% answered “no”). This did not differ across gender, age, or socioeconomic groups. Reasons for concern were given by a small number of respondents (15/111, 13.5%), with privacy and security most common (10/15). Other reasons for individual concern included accuracy of information (2/15), computer literacy and access (2/15), limited Australian-based sites (1/15), the opportunity for misinterpretation of information (1/15), and the lack of a support group for that individual’s particular condition (1/15) (Table 3).

## Discussion

### Principal Findings

Web-based health information and support are available in the United States [28, 32-35]; however, internationally, the use is not as widespread [28,30-36]. In the Australian context, studies have explored Web-based health interventions, but there is limited information as to the patterns of technology use in cardiopulmonary patients [37]. The reported study sought to describe patterns of technology use in patients attending a cardiopulmonary clinic. The patients responding to this survey provide a sample of those individuals likely to have increasing health care utilization needs. It is important to take this initial step in understanding whether these consumers have technology capabilities and receptivity to these modalities [42-44].

Study results indicated that computer, mobile phone, and Internet use are a pervasive part of everyday life with individuals using their technological devices for a variety of reasons, including accessing and browsing health information websites. The majority of respondents additionally answered that access to support programs through a telecommunication platform would provide assistance with their health problems; this was most common in individuals aged 50-65 years. The most commonly accessed websites were disease-specific sites, organizations, and research sites, as well as sites that provided information on specific medications and treatments. In agreement with previous literature, peer support and sharing of experiences were also noted as benefits of access through an online platform, and was noted as providing support and information that they may not otherwise be able to access in their everyday life [29-31,46,47].

Patterns of technology use did differ between patients from different socioeconomic groups, as measured using advantage/disadvantage index based on area [45]. Although overall technology use was pervasive in all groups, patients who lived in higher socioeconomic areas used the Internet most regularly and those patients from middle socioeconomic areas were most likely to access social media compared with the other groups. Although still high users of technology, those from low socioeconomic areas had less access to computers and used computers and the Internet less frequently, a situation evident in international literature; technological access and literacy are a consideration for future technology-based health delivery interventions [48,49]. Interestingly, while those who lived in higher socioeconomic areas were most likely to be already accessing Web-based health information sites, when asked if they would benefit from delivery of health information and support through an online interface, those from higher socioeconomic areas were least likely to respond positively; this may reflect higher health literacy [27,50], better health access [2,45], and therefore less need for additional support, but this would need further investigation.

Respondents indicated clear issues with current health care delivery through face-to-face interaction, with several noting the long distance they had to travel to access care and the lack of information provision through traditional sources; Web-based health information delivery may go some way to alleviating the limitations of current health care provision. As similarly noted in previous literature, respondents viewed online health care delivery as providing convenient, timely, and easily accessible information, currently difficult to obtain through traditional face-to-face sources [29-31,51]. This is particularly important in the context of this cardiopulmonary clinic, which acts as a quaternary referral clinic attracting patients from a large geographical area across the state. Several studies have highlighted the relationship between patient satisfaction and Web-based health information seeking behavior [51-53]. Consumer-health provider interfaces need to be improved to provide timely and accessible health care interaction that reduces the geographical burden of current health care delivery [28-31,51-53].

While the majority of respondents stated that they did not have concerns over accessing information or support online, issues of privacy and security, the accuracy of information, and the potential for misinterpretation of information were raised by a smaller number of patients. Consumers’ ability to distinguish accurate, trustworthy, and personally applicable information, when faced with the sheer volume of health information sites available, is a commonly reported challenge in the literature [42-44]. Development and validation of websites is essential; health professionals have an opportunity to ensure that patients and their families have guidance to accurate and trustworthy Web-based health information sources [42-44].

Web-based health care delivery has particular potential to provide convenient and accessible access for individuals and their families living with chronic, complex, and progressive conditions [28-31]. Providing ongoing care through technology platforms may address the issues associated with short-term episodic programs, such as pulmonary and cardiac rehabilitation, in providing ongoing education, social support, and exercise maintenance to larger patient cohorts [16,17,21,22,28]. Self-management programs that are provided through a Web-based interface may leverage computer-based and mobile technology to facilitate continued care and support [28-31]. This may be of particular value to aging “baby boomers”, who have already incorporated these technologies into their daily lives [28-31]. Web-based health care delivery additionally has the potential to help those at end of life who need increasingly complex strategies to cope with dyspnea and fatigue, especially as they become homebound [28-31].

### Implications for Practice

This study sought to describe patterns of technology use in patients attending a cardiopulmonary clinic. Technology use is a pervasive part of everyday life regardless of age or socioeconomic group with patients already heavily engaged in health-seeking behaviors through Web-based sources. There is a necessity to develop and validate websites, and an opportunity to ensure that patients and their families have guidance to accurate and credible health information sources. Web-based delivery of health information and support is of particular importance in patients with cardiopulmonary disease, who are most likely to have high symptom burden and health care utilization needs in the future. Current consumer-health provider interfaces need to be improved to accommodate the changing needs and preferences of an empowered generation of health consumers, and to provide timely and accessible health care interaction that addresses the geographical burden of current health care delivery. Telecommunication interfaces may alleviate some of the difficulties with current health care access and provide an increasingly important adjunct to traditional forms of health care delivery. We are at a turning point within the evolution of health care delivery and have the opportunity to shape how future interventions deliver health information and promote self-management. Future research must explore the feasibility of delivering health care through Web-based platforms across larger cohorts and explore the social and economic impact of this approach on health care delivery.

### Limitations

This prevalence study was undertaken in a small cohort of patients from a single clinical site. While survey responses were completed in full in most cases, there is a possibility that patients who do not engage with technology may have self-excluded from participating. Further, large cohort, multi-site research would be required to describe overall population technology use. Additionally, this study only sought to describe the patterns of technology use and further research is required to understand the attitudes and specific barriers faced by cardiopulmonary pulmonary patients in regard to the delivery of health information and education through telecommunication interfaces. While this initial study does have its limitations, the results do provide important information regarding patients’ access to technological devices, their use of Web-based information and support for their health conditions, and the perceived potential benefits of health care delivery through Web-based platforms. This is particularly important in the context of patients attending a cardiopulmonary clinic, who are most likely to have high symptom burden and associated health care utilization needs in the future.

### Conclusions

Chronic disease burden and the long-term self-management tasks that challenge patients with cardiopulmonary disease are a compelling argument for accessible and convenient avenues to obtaining ongoing treatment and peer support. Technology use was already a pervasive part of everyday life for study participants, and a central platform for health care interactions including common access of health information and education. Patterns of use and access differed marginally across age and socioeconomic groups, however, accessing Web-based health information was prevalent for all groups. Clear issues were raised over long distance travel and a lack of information provision through traditional health delivery sources. Web-based access to health support programs are perceived as providing convenient, timely, and easily accessible information—particularly important in the context of a quaternary referral clinic attracting patients from a large geographical area, and in cardiopulmonary patients most likely to have high health care utilization needs in the future. Telecommunication interfaces will be an increasingly important adjunct to traditional forms of health care delivery. These will need to be assessed for the validity of content and access to target populations.

## Multimedia Appendix 1

Internet access and use survey.

## Abbreviations

CHF: congestive heart failure
COPD: chronic obstructive pulmonary disease
