# Supplementary material for: Patterns of Technology Use in Patients Attending a Cardiopulmonary Outpatient Clinic: A Self-Report Survey
Source: Interact J Med Res. 2015 Mar 6;4(1):e5. doi: 10.2196/ijmr.3955 (PMC4376160; doi:10.2196/ijmr.3955)
Supplement: Supplementary file 1 [file ijmr_v4i1e5_app1.pdf]

Multimedia Appendix 1

INTERNET ACCESS AND USE SURVEY

Demographics

Male Female

Postcode ___________

Date of birth __/__/_____

Section 1 Computer access

1. Do you use a computer regularly? (more than 4 times a week)

Yes No

2. Do you have access to the following **at home** for personal use?

(please tick all that apply)

Desktop computer

Laptop

Tablet computer (i.e. iPad or similar)

3. Do you have access to the following **at work** for personal use?

Computer

Laptop

Tablet computer (i.e. iPad or similar)

Section 2 Internet access

4. Do you use the **internet** regularly? (more than 4 times a week)

Yes No

5. Do you have internet access at **home**?

Yes No

If yes, is this:

Broadband

Dial-up

Cable/DSL/fibre

Wireless

Unsure

6. Is there anywhere else where you **access** the internet regularly?

(please tick all that apply)

Via a smartphone

Wireless connection in public places (i.e. cafe's etc)

Internet cafe

Public library or educational institution

Friend or family's place

Other ________________________

7. What activities do you use the internet for?

(please tick all that apply)

Emailing
Social media (Facebook etc)
Skype or video calls
Shopping
Browsing, researching, reading news articles
Browsing for health information
Other ______________________

Section 3 Mobile phone access

8. Do you own a mobile phone?

Yes No

If yes, what do you use your mobile phone for? (tick all that apply)

Phone calls
Sending SMS texts
Checking /sending emails
Internet browsing
Other ___________________

Section 4 Health support programs via the internet

9. Are there health education or social group sites on the internet that you have found helpful?

Yes No

If yes, please provide examples:_________________________________

10. Would you find it useful to be able to access support programs using the internet to assist you with your health problems?

Yes No

Why? ____

11. Would you have any concerns about participating in support programs via the internet?

Yes No

Why? ____

Thank you for taking the time to participate in this study.
